# Associations among autistic traits, cognitive and affective empathy, and personality traits in adults with autism spectrum disorder and no intellectual disability

**DOI:** 10.1038/s41598-022-07101-x

**Published:** 2022-02-24

**Authors:** Yukihiko Shirayama, Kazuki Matsumoto, Sayo Hamatani, Katsumasa Muneoka, Akihiro Okada, Koichi Sato

**Affiliations:** 1 Department of Psychiatry, Teikyo University Chiba Medical Center, 3426-3 Anesaki, Ichihara, 299-0111 Japan; 2 Department of Psychology, Sapporo International University, Sapporo, Japan

**Keywords:** Psychiatric disorders, Translational research

## Abstract

Reported empathy deficits in autism spectrum disorder (ASD) could be attributable to other ASD-related features. We evaluated 28 ASD adults with no intellectual disability and 24 age-matched non-ASD control subjects using the Autism-Spectrum Quotient (AQ), Questionnaire of Cognitive and Affective Empathy (QCAE), Interpersonal Reactivity Index (IRI), and NEO Personality Inventory-Revised (NEO). Compared to the controls, ASD participants showed lower scores for perspective taking, online simulation, cognitive empathy, and peripheral responsivity on the QCAE, and lower scores for perspective taking and empathic concern on the IRI. Within the ASD group, the AQ scores showed significant relationships with perspective taking, online simulation and cognitive empathy on the QCAE, and perspective taking on the IRI. The ASD group also showed higher scores for neuroticism and lower scores for extraversion on the NEO compared to the controls. However, there were no relationships between AQ scores and NEO factors within the ASD group. Multiple regression analysis with stepwise linear regression demonstrated that perspective taking score on the QCAE and extraversion score on the NEO were good predictor variables to autistic traits on the AQ. These findings help us to understand empathy and personality traits in ASD adults with no intellectual disability.

## Introduction

People with autism spectrum disorder (ASD) typically have problems with reciprocal social interaction and communication and show restricted interests. As a result, they can experience challenges that interfere with their activities of daily living. It seems that individuals with ASD often have troubles with social perception and competence due to difficulties in reading nonverbal interactive cues and responding typically in conversations.

In the Diagnostic and Statistical Manual of Mental Disorders, 5th Edition (DSM-5)^1^, Asperger syndrome and high-functioning ASD have been rolled into the single category “ASD”, with a note to specify language or intellectual impairment or associated conditions (e.g., genetic or medical diagnosis). Asperger syndrome was once distinguished from other ASD conditions by an association with typical early language development, and individuals with intelligence quotient (IQ) scores within or above the normal average range have been described as “high-functioning”.

Some researchers have suggested that autism is linked to an innate impairment in the ability to perceive and respond to the affective expressions, leading to profound difficulties in social interaction. Impairments in empathy were reported in ASD individuals with normal IQ^2,3^. Empathy involves cognitive and affective components. Cognitive empathy is defined as the capacity to understand other people’s feelings, intentions, and beliefs on an intellectual level, while affective empathy is the emotional response to other people’s affective state or feelings^4,5^. Some researchers have reported atypical theory of mind in adolescents and adults with ASD^6–8^. Empathy assessment using the Interpersonal Reactivity Index (IRI)^4^ demonstrated that ASD adults with no intellectual disability showed impairments in perspective taking scale^3,8–11^, suggesting potential difficulties in representing another person’s psychological perspective. A previous study highlighted that autism is related to cognitive empathy deficits but not for affective empathy^12^.

Adults with ASD might well have acquired personality bias. Investigators found that adults with what was once called Asperger syndrome showed elevated harm avoidance and low self-directedness and cooperativeness on the Temperament and Character Inventory (TCI)^13–16^. Self-directedness and cooperativeness are addressing maturity of personality from the perspective of self and others. The subscales of cooperativeness include social acceptance, empathy, helpfulness and pure-heartedness. Another personality inventory, the NEO Personality Inventory-Revised (NEO)^17^ is a five-factor model of personality structures in terms of five traits: neuroticism, extraversion, openness, agreeableness, and conscientiousness. Adults with ASD showed high neuroticism and low extraversion, openness, agreeableness and conscientiousness on the NEO^18,19^. Although the personality traits of neuroticism and extraversion are associated with negative and positive emotional experiences, respectively^20^, the relationships between the personality traits and autistic traits in ASD are not well documented.

In clinical situations, adults with ASD have personality traits. However, it is unknown whether the autistic behaviors in ASD are related with empathy deficits and personality traits. Here, we hypothesized that empathy deficits and personality traits are constituent parts of autistic behaviors in ASD. To test this hypothesis, we examine what types of components exist in empathy deficits and personality traits and modulate autistic behaviors in ASD adults with no intellectual disability. First, we extracted important components in empathy deficits and personality traits in ASD. Second, we examined the relationships among autistic traits, empathy and personality in each group and compared them. Finally, we quantitatively conducted multiple regression analysis to examine the contribution of empathy and personality traits to the autistic traits.

## Methods

### Participants

This study included 24 ASD adults with no intellectual disability and 28 non-ASD control adults. Inclusion criteria required all participants to be drug-naïve. Participating adults with ASD were recruited from the outpatient clinic of Teikyo University Chiba Medical Center, and all met the DSM-5 criteria for ASD, requiring consensus based on more than 4 months of longitudinal follow-up examination by trained psychiatrists and psychologists. Exclusion criteria were a history of head trauma, seizures or other neurological disorders, intellectual disability, or alcohol and substance use disorders. The adults with ASD had no other psychiatric disorders including depression at enrollment. Non-ASD control subjects with no past history of psychiatric disorders or drug dependence were recruited within the social environment of the authors.

Participant characteristics are shown in Table 1. Adults with ASD scored significantly higher for autistic traits (Autism-Spectrum Quotient, AQ)^21^ compared with healthy controls (*p* < 0.001) (Table 1). Also, adults with ASD showed statistical significance on the Autism Diagnostic Observation Schedule, second edition (ADOS-2) for clinical use^22^. The depressive level on the Beck Depression Inventory score (BDI)^23^ was significantly higher for the adults with ASD compared with unaffected controls (*p* = 0.012) (Table 1). Full IQ was significantly different between the two groups (*p* = 0.016) (Table 1), but the IQ of all the subjects were within normal range (IQ > 80).

**Table 1:** The participant demographics.

	Non-ASD control (n = 28)	ASD (n = 24)	*p*-values
Age, years (range)	30.4 ± 6.2 (23–44)	27.5 ± 7.5 (18–44)	0.144
Gender (male/female)	12/16	14/10	0.163
AQ	17.3 ± 6.4	32.7 ± 6.5	< 0.001***
ADOS-2 for clinical use	0.5 ± 1.7	7.13 ± 2.7	< 0.001***
BDI	5.3 ± 6.4	10.0 ± 6.5	0.012*
Full IQ	106.2 ± 13.7	97.2 ± 11.7	0.016*
Verbal IQ	106.4 ± 13.1	98.4 ± 13.4	0.033*
Performance IQ	104.9 ± 14.1	96.5 ± 13.8	0.036*

Data are mean ± SD.

*AQ* autism spectrum quotient, *ADOS-2* autism diagnostic observation schedule, 2nd edition, *BDI* Beck depression inventory, *IQ* intelligence quotient.

*p < 0.05,**p < 0.01,***p < 0.001 compared to the non-ASD controls (Student’s t-test).

### Instruments

The Autism-Spectrum Quotient (AQ), measuring autistic traits^21^, is a 50-item self-administered screening questionnaire to identify the degree to which adults of average intelligence might have autism-related features. This test covers five areas: social skills, attention switching, attention to detail, communication, and imagination. The cutoff for ASD is set at > 32, which captures 80% of adult ASD individuals with no intellectual disability; only 2% of unaffected adults surpass this cutoff. Another study reported that the AQ has good discriminative validity and screening properties with a threshold score of 26^24^. Internal consistency reliability by Cronbach' alpha was 0.808.

Questionnaire of Cognitive and Affective Empathy (QCAE), which consist of 31 items, comprising five subscales: perspective taking, online simulation, emotion contagion, proximal responsivity, and peripheral responsivity, was used to assess level of cognitive and affective empathy^25^. The cognitive empathy subcomponents are perspective taking and online simulation, and the affective empathy subcomponents are emotion contagion, proximal responsivity, and peripheral responsivity. The perspective taking scale reflects placing oneself intuitively in another person’s shoes. The online simulation component is a more effortful process of attempting to understand the emotional states of others, whereas the emotion contagion reflects the automaticity of mirroring the emotional states of others. Proximal responsivity is the responsiveness of affective situations in a close social context, and peripheral responsivity is the responsiveness to affective situations that occur in a more detached context.

The Interpersonal Reactivity Index (IRI) was used to assess empathy. The IRI consists of 28 items comprising four subscales: perspective taking, fantasy, empathic concern, and personal distress^4^. The perspective taking scale assesses ability to arrive at a cognitive understanding of what another person thinks or feels. The empathic concern scale reflects the tendency to feel emotional compassion and concern for unfortunate others. The personal distress scale measures self-oriented feelings of anxiety and discomfort in response to other people’s suffering. The fantasy scale assesses a person’s ability to self-project into fictional situations.

Personality was assessed using the NEO Personality Inventory-Revised (NEO), which relies on the five-factor model of personality: neuroticism, extraversion, openness, agreeableness, and conscientiousness^17^. It consists of 240 items answered on a five-point Likert scale. The mean and SD for each dimension are 50 and 10, respectively. The neuroticism scale identifies individuals who are prone to psychological distress. The extraversion scale refers to individuals who are sociable, communicative and prone to adventure and simulation. The openness scale identifies individuals who are open to new ideas and are unconventional in the set of values. The agreeableness scale assesses the type of interaction individuals prefer from compassionate to tough mindedness. The conscientiousness scale assesses the degree of organization, persistence, control, and motivation in goal-directed behavior.

Autism Diagnostic Observation Schedule, second edition (ADOS-2) for clinical use was done to check the ASD character^22^. Beck Depression Inventory score (BDI) was used for assessing depression features^23^. IQ scores were estimated using the Wechsler Adult Intelligence Scale, 3rd edition (WAIS-III)^26^.

### Statistical analysis

We began using multiple analysis of variance (MANOVA) to analyze data from five domains of QCAE and four domains of IRI to check for the simultaneous significant differences between the two groups. Additional covariate analysis was performed using analysis of covariance (ANCOVA), treating BDI and full-scale IQ scores as covariates. Homogeneity of variance was checked by Box's M test and Levene's test. Multicollinearity was checked by multiple regression analysis with stepwise linear method. Multivariate normality and a linear relationship between dependent variables for the independent variable were checked by histogram of residual differences and a scatter P-P plot. Model fit was checked by lack of fit tests.

Coefficients of AQ scores with QCAE or IRI scores, and NEO factors were estimated within each group by Pearson coefficients. Effects size was calculated using G-power (3.1). Bonferroni correction for multiple comparisons was used when appropriate (QCAE seven scores and IRI four scores, *p* < 0.05/28 = 0.0018) (QCAE + IRI eleven scores and NEO five scores, *p* < 0.05/55 = 0.0009).

Multiple regression analysis with stepwise linear regression was conducted, treating AQ scores as the dependent variable, and, QCAE five subscales (perspective taking, online simulation, emotion contagion, proximal responsivity, peripheral responsivity), IRI four subscales (perspective taking, empathic concern, personal distress, fantasy), and NEO five subscales (neuroticism, extraversion, openness, agreeableness, conscientiousness) as independent variables. A better good-fit model was determined by standardized β, adjusted R Square (R^2^) and F value.

Multiple group structural equation modeling was conducted to examine comparison of patterns of contributing two factors (perspective taking on the QCAE and extraversion on the NEO) to AQ between non-ASD control and ASD groups.

Effects size was calculated using G-power (3.1). Internal consistency reliabilities were expressed by Cronbach' alpha (Supplementary Table S1). Collinearities of variables were checked by variance inflation factor (Supplementary Table S2). Comparison of correlations between two groups was conducted using cocor R package (alpha = 0.05, confidence level = 0.95, null value = 0). Chi-square test was used for categorical variables. Differences were set as significant at *p* < 0.05. Analyses were conducted with SPSS version 20 (IBM) and SPSS AMOS version 28 (IBM).

### Ethics declarations

This research was approved by the ethics committee of Teikyo University School of Medicine (ethical committee approval No.17-105) and performed in accordance with the Declaration of Helsinki. Written informed consent was obtained after the procedures had been fully explained to each participant.

## Results

### Empathy measures

For the QCAE, MANOVA for the five domains indicated a significant group effect (F = 7.425, *p* < 0.001), demonstrating that participants with ASD had significantly lower scores for perspective taking, online simulation and peripheral responsivity, but not for emotion contagion or proximal responsivity (Table 2). After controlling for the full-scale IQ and BDI values by ANCOVA, significant differences remained for perspective taking, online simulation and peripheral responsivity (Table 2). When we combined the subcategory data into the two categories on the QCAE, *t*-tests showed that adults with ASD had significantly lower scores for cognitive empathy, but not for affective empathy (Table 2). After adjustment for full-scale IQ and BDI values by ANCOVA, the difference in cognitive empathy remained significant, but not in affective empathy (Table 2). When Bonferroni corrections were done for these results, the results were statistically significant (p < 0.05/7 = 0.007).

**Table 2:** Empathy data on the QCAE and IRI.

	Non-ASD control (n = 28) [95% CI]	ASD (n = 24) [95% CI]	*p*-values (uncorrected)	*p*-values with cofactors	η^2^	Effect size
QCAE Perspective taking	35.0 ± 6.4 [32.5–38.2]	24.3 ± 7.7 [20.8–27.0]	< 0.001***	< 0.001***	0.364	1.511
QCAE Online simulation	34.1 ± 5.1 [31.5–36.6]	26.5 ± 7.6 [23.7–29.3]	< 0.001***	< 0.001***	0.232	1.174
QCAE Emotion contagion	13.1 ± 2.8 [11.7–14.6]	13.2 ± 4.3 [11.6–14.7]	0.919	0.950	0.000	0.027
QCAE Proximal responsivity	12.0 ± 2.6 [10.7–13.1]	10.7 ± 3.3 [9.4–12.0]	0.111	0.185	0.036	0.437
QCAE Peripheral responsivity	14.2 ± 2.4 [13.3–15.6]	11.9 ± 3.2 [10.3–12.7]	0.005**	0.001**	0.194	0.813
QCAE Cognitive empathy	69.0 ± 10.8 [64.5 – 74.3]	50.6 ± 13.4 [44.9 – 55.6]	< 0.001***	< 0.001***	0.346	1.512
QCAE Affective empathy	39.3 ± 6.2 [36.7 – 42.3]	35.7 ± 8.0 [32.4 – 38.5]	0.079	0.067	0.068	0.503
IRI Perspective taking	21.2 ± 2.8 [19.8–22.5]	16.6 ± 3.6 [15.3–18.1]	< 0.001***	< 0.001***	0.292	1.426
IRI Empathic concern	20.7 ± 3.2 [19.7–21.9]	18.7 ± 2.4 [17.3–19.7]	0.010*	0.010*	0.130	0.707
IRI Personal distress	17.5 ± 4.0 [15.9–19.4]	19.3 ± 4.6 [17.3–21.1]	0.147	0.247	0.028	0.417
IRI Fantasy	19.8 ± 3.7 [18.3–21.5]	17.7 ± 4.4 [15.7–19.2]	0.067	0.059	0.072	0.516

Data are mean ± SD. The number in the bracket is 95% confidence intervals (CI).

*η*^*2*^ semi-partial eta-squared. Effect size represents a sample-based estimate of the quality, *QCAE* Questionnaire of Cognitive and Affective Empathy, *IRI* Interpersonal Reactivity Index.

*p < 0.05,**p < 0.01,***p < 0.001. Uncorrected *p*-values are determined by MANOVA followed by *t*-tests. Corrected *p*-values were obtained by a subsequent ANCOVA treating the BDI ad full-scale IQ scores as covariates.

For the IRI, MANOVA of data for the four domains indicated a significant group effect (F = 7.829, *p* < 0.001), showing that the adults with ASD had significantly lower scores for perspective taking and empathic concern, but not for personal distress or fantasy (Table 2). After controlling for full-scale IQ and BDI scores by ANCOVA, significant differences remained only for perspective taking and empathic concern (Table 2). When Bonferroni corrections were done for these results, the results were statistically significant (p < 0.05/4 = 0.012).

### The correlations of ASD traits with empathy scores on the QCAE and IRI

**Figure 1:**
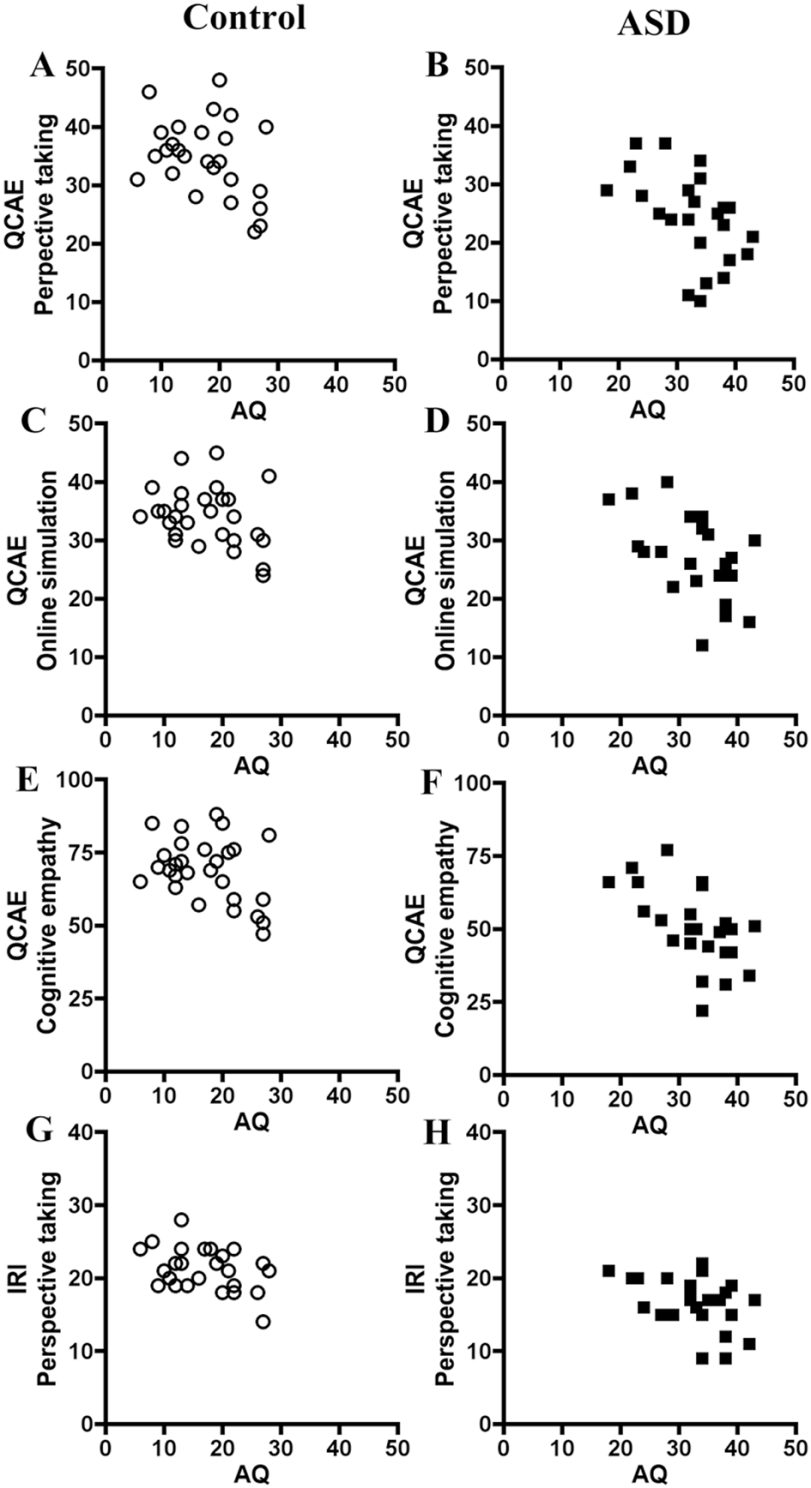
The correlation plots for the association between autistic traits and empathy. **(A,B)** The correlations between autistic traits on the AQ and perspective taking on the QCAE in the control and ASD subjects. **(C,D)** The correlations between autistic traits on the AQ and online simulation on the QCAE in the control and ASD subjects. **(E,F)** The correlations between autistic traits on the AQ and cognitive empathy on the QCAE in the control and ASD subjects. **(G,H)** The correlations between autistic traits on the AQ and perspective taking on the IRI in the control and ASD subjects.

Total AQ scores correlated significantly with the perspective taking and cognitive empathy scores on the QCAE, within control group and ASD group, respectively (Table 3). Additionally, total AQ scores correlated significantly with the online simulation scores on the QCAE and the perspective taking scores on the IRI only among ASD group (Table 3). However, total AQ scores failed to show significant relationships with the peripheral responsivity on the QCAE and the empathic concern on the IRI among each group in spite of significance of group comparison (Tables 2, 3). When Bonferroni corrections were done for these results, the correlation between AQ scores and cognitive empathy on the QCAE within ASD group was statistically significant (p < 0.05/7 = 0.007). The correlation plots for key results (perspective taking, online simulation, and cognitive empathy on the QCAE and perspective taking on the IRI) were shown in Fig. 1.

**Table 3:** Relationships between empathy's components on the QCAE and IRI and autistic traits on the AQ.

	AQ	IRI Perspective taking	IRI Empathic concern	IRI Personal distress	IRI Fantasy
**Non-ASD control (n = 28)**					
AQ		−0.315	0.056	0.301	−0.054
QCAE Perspective taking	−0.387*	0.597**	0.136	−0.186	0.113
QCAE Online simulation	−0.299	0.669***	0.214	−0.227	0.163
QCAE Emotional contagion	−0.002	0.011	0.281	0.158	0.335
QCAE Proximal responsivity	−0.068	−0.064	0.395* C	0.166	0.279
QCAE Peripheral responsivity	−0.361	0.010	0.443* C	0.069	0.561**
QCAE Cognitive empathy	−0.375*	0.670***	0.182	-0.215	0.148
QCAE Affective empathy	−0.172	0.007	0.468* C	0.168	0.490** C
**ASD subjects (n = 24)**					
AQ		−0.430* A	−0.078	0.308	−0.064
QCAE Perspective taking	−0.510*	0.581**	0.079	−0.327	−0.066
QCAE Online simulation	−0.499* A	0.821***	-0.115	−0.292	−0.030
QCAE Emotional contagion	0.098	0.095	0.528** A	0.576** A	0.223
QCAE Proximal responsivity	0.010	0.290	0.294	0.555** A	0.074
QCAE Peripheral responsivity	−0.085	0.007	−0.028	−0.170	0.541**
QCAE Cognitive empathy	−0.579**	0.791***	−0.019	−0.355	−0.057
QCAE Affective empathy	0.022	0.174	0.394	0.471* A	0.370

Values are Pearson's r correlations.

*AQ* autism-spectrum quotient, *IRI* Interpersonal Reactivity Index, *QCAE* Questionnaire of Cognitive and Affective Empathy, *C* only within non-ASD control subjects, *A* only within ASD subjects.

*p < 0.05,**p < 0.01,***p < 0.001 in each group.

### The relationships among empathy scores on the QCAE and IRI

The purpose of the correlation analyses between subscales in the QCAE and subscales in the IRI was to examine the difference and relations between the items for empathic traits on the two questionnaires within non-ASD control and ASD groups.

As for empathy scores, we found strong correlations of the perspective taking scores on the IRI with the perspective taking, online simulation and cognitive empathy scores on the QCAE for controls and ASD group, respectively (Table 3). This replicated a previous report in control subjects^27^. Also we found significant correlations between peripheral responsivity on the QCAE and fantasy scores on the IRI for control group and for ASD group (Table 3). When Bonferroni corrections were done for these results, the correlations between online simulation and cognitive empathy on the QCAE and perspective taking on the IRI were statistically significant (p < 0.05/28 = 0.0014).

On the contrary, we found different patterns between the two groups. Among control group there existed correlations between the empathic concern scores on the IRI and the proximal responsivity, peripheral responsivity and affective empathy on the QCAE in addition to between fantasy scores on the IRI and affective scores on the QCAE (Table 3), whereas among ASD group there existed correlations between the empathic concern scores on the IRI and emotional contagion scores on the QCAE in addition to between the personal distress scores on the IRI and emotional contagion, proximal responsivity and affective empathy scores on the QCAE (Table 3). Comparison of correlations by cocor demonstrated significant differences in the correlations between the online simulation on the QCAE and perspective taking on the IRI, the emotional contagion on the QCAE and personal distress on the IRI, and the peripheral responsivity on the QCAE and empathic concern on the IRI between the two groups. However, when Bonferroni corrections were done for these results, correlations were not statistically significant (p < 0.05/20 = 0.0025).

### Personality scores

For the NEO, MANOVA indicated a significant group effect (F = 8.951, *p* < 0.001), demonstrating that compared with control subjects, the adults with ASD had significantly higher scores for neuroticism and lower scores for extraversion, agreeableness and conscientiousness, but no differences in openness, on the NEO, (Table 4). After controlling for the full scale IQ and BDI scales by ANCOVA, significant differences remained in neuroticism and extraversion, but not in agreeableness or conscientiousness (Table 4). When Bonferroni corrections were done for these results, the results were statistically significant (p < 0.05/5 = 0.01).

**Table 4:** Personality data on the NEO.

	Non-ASD control (n = 28) [95% CI]	ASD (n = 24) [95% CI]	*p*-values (uncorrected)	*p*-values with cofactors	η^2^	Effect size
Neuroticism	55.2 ± 10.4 [52.6–60.7]	67.8 ± 10.7 [61.6–70.4]	< 0.001***	0.004**	0.158	1.194
Extraversion	49.8 ± 11.9 [45.2–53.3]	34.7 ± 8.7 [30.9–39.8]	< 0.001***	< 0.001***	0.290	1.448
Openness	51.9 ± 7.1 [48.6–55.2]	49.4 ± 9.4 [45.8–53.0]	0.271	0.338	0.019	0.300
Agreeableness	48.1 ± 11.6 [42.7–52.4]	40.6 ± 12.4 [35.9–46.5]	0.028*	0.098	0.056	0.619
Conscientiousness	45.6 ± 10.3 [40.1–48.6]	36.6 ± 11.4 [33.5–42.7]	0.004**	0.065	0.069	0.828

Data are mean ± SD. The number in the bracket is the value of 95% confidence intervals (CI).

*η*^*2*^ semi-partial eta-squared. Effect size represents a sample-based estimate of the quality. *NEO* NEO Personality Inventory-Revised.

*p < 0.05,**p < 0.01,***p < 0.001. Uncorrected *p*-values are determined by MANOVA followed by *t*-tests. Corrected *p* values were done by subsequent ANCOVA, treating BDI ad full-scale IQ scores as covariates.

### Correlations of AQ scores with NEO personality scores

**Figure 2:**
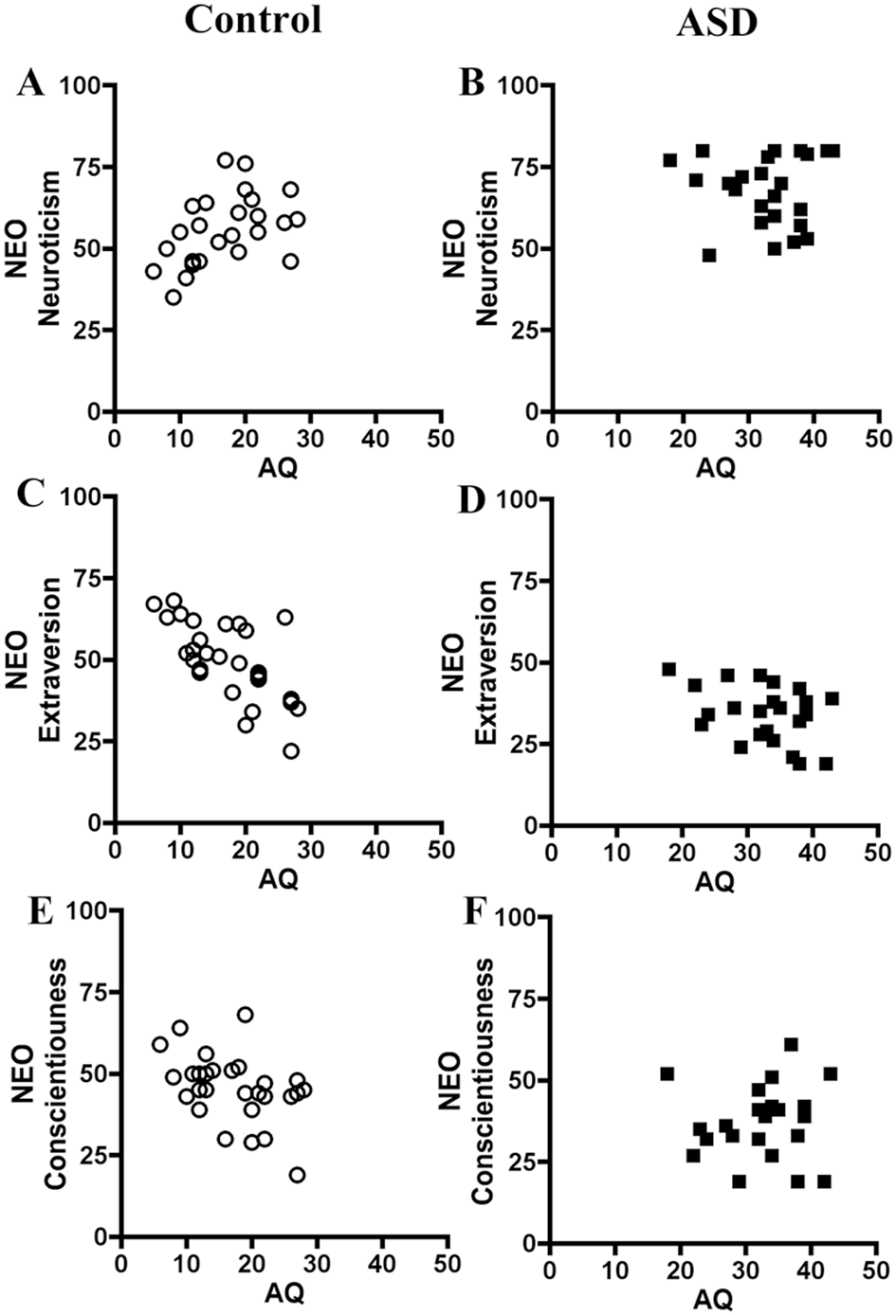
The correlation plots for the association between autistic traits and personality. **(A,B)** The correlations between autistic traits on the AQ and neuroticism on the NEO in the control and ASD subjects. **(C,D)** The correlations between autistic traits on the AQ and extraversion on the NEO in the control and ASD subjects. **(E,F)** The correlations between autistic traits on the AQ and conscientiousness on the NEO in the control and ASD subjects.

We found significant correlations between AQ scores and NEO personality scores: for neuroticism among non-ASD controls and total participants, but not among ASD group; for extraversion among healthy controls and total participants, but not among ASD group; for conscientiousness among healthy controls and total participants, but not among ASD group (Table 5). When Bonferroni corrections were done for these results, the correlations between AQ scores and the neuroticism, extraversion and conscientiousness on the NEO within total subjects were statistically significant (p < 0.05/5 = 0.01). The correlation plots for key results (neuroticism, extraversion and conscientiousness on the NEO) were shown in Fig. 2.

**Table 5:** Correlations between AQ scores and NEO categories.

	AQ Non-ASD control (n = 28)	AQ ASD subjects (n = 24)	AQ Total participants (n = 52)
Neuroticism	0.404*	−0.015	0.514*
Extraversion	−0.668***	−0.357	−0.733***
Openness	0.185	−0.160	−0.120
Agreeableness	0.118	0.013	−0.194
Conscientiousness	−0.435*	−0.010	−0.433**

Values are Pearson's r correlations.

*p < 0.05,**p < 0.01,***p < 0.001 in each group.

*AQ* autism-spectrum quotient, *NEO* NEO Personality Inventory-Revised.

### Relationships of personality factors on the NEO with empathy scales on the QCAE and IRI

Within each control group and ASD group, we found significant correlations of neuroticism on the NEO with emotion contagion and affective empathy on the QCAE, and with fantasy on the IRI (Table 6). Further, we found significant correlations of extraversion on the NEO with peripheral responsivity on the QCAE, and of openness on the NEO with peripheral responsivity on the QCAE within each group (Table 6).

**Table 6:** Correlations between empathy and personality.

	NEO Neuroticism	NEO Extraversion	NEO Openness	NEO Agreeableness	NEO Conscientiousness
**Non-ASD control (n = 28)**					
QCAE Perspective taking	0.203	0.041	0.000	0.017	0.165
QCAE Online simulation	0.095	0.188	0.228	0.133	0.342
QCAE Emotion contagion	0.522**	0.101	0.370	0.142	-0.247
QCAE Proximal responsivity	0.427* C	0.236	0.389* C	0.221	0.058
QCAE Peripheral responsivity	0.197	0.499**	0.531**	0.096	0.047
QCAE Cognitive empathy	0.165	0.114	0.110	0.075	0.258
QCAE Affective empathy	0.492**	0.341	0.540** C	-0.008	-0.068
IRI Perspective taking	0.030	0.001	−0.003	0.186	0.192
IRI Empathic concern	0.387* C	−0.065	0.321	0.141	0.022
IRI Personal distress	0.262	−0.320	0.169	0.292	−0.472* C
IRI Fantasy	0.480**	0.168	0.343	0.015	−0.191
**ASD (n = 24)**					
QCAE Perspective taking	−0.081	−0.097	0.209	0.082	0.233
QCAE Online simulation	−0.159	0.444* A	0.480* A	0.087	0.354
QCAE Emotion contagion	0.446*	0.185	−0.198	0.142	−0.232
QCAE Proximal responsivity	0.327	0.324	0.074	0.229	0.200
QCAE Peripheral responsivity	0.262	0.490*	0.501*	−0.403	−0.268
QCAE Cognitive empathy	−0.127	0.194	0.388	0.090	0.330
QCAE Affective empathy	0.481*	0.432* A	0.127	0.008	−0.151
IRI Perspective taking	-0.349	0.156	0.388	0.185	0.511* A
IRI Empathic concern	0.328	0.299	−0.144	0.234	−0.252
IRI Personal distress	0.486* A	−0.118	0.216	0.328	−0.245
IRI Fantasy	0.447*	0.248	0.159	−0.378	−0.253

Values are Pearson's r correlations.

*IRI* Interpersonal Reactivity Index, *NEO* NEO Personality Inventory-Revised, *QCAE* Questionnaire of Cognitive and Affective Empathy, *C* only within control subjects, *A* only within ASD patients.

*p < 0.05,**p < 0.01, coefficient in each group.

Within only control group, there existed significant correlations between neuroticism on the NEO and proxismal responsivity on the QCAE, openness on the NEO and proxismal responsivity and affective empathy on the QCAE, and conscientiousness on the NEO and personal distress on the IRI (Table 6).

Within only ASD group, we found significant correlations between neuroticism on the NEO and personal distress on the IRI, extraversion on the NEO and online simulation on the QCAE, extraversion on the NEO and affective empathy on the QCAE, openness on the NEO and online simulation on the QCAE, and conscientiousness on the NEO and perspective taking on the IRI (Table 6).

When Bonferroni corrections were done for these results, correlations were not statistically significant (*p* < 0.05/55 = 0.0009).

Comparison of correlations by cocor failed to demonstrate significant differences in the correlations between personality factors and empathy scales between the two groups.

### Contributory factors of empathy and personality to autistic traits by multiple regression analysis

Multiple regression analysis with stepwise linear regression showed goodness-of-fit statistics (R = 0.872, adjusted R^2^ = 0.750, F = 77.55, *p* < 0.001). Results indicated that two factors, extraversion on the NEO (standardized coefficient beta = −0.556, *p* < 0.001) and perspective taking on the QCAE (standardized coefficient beta = −0.504, *p* < 0.001), were good predictor variables to autistic traits on the AQ scores (Supplementary Table S2).

Next, multiple-group structural equation modeling was conducted. This model had good fit and showed that standardized coefficients were -0.659 (*p* < 0.001) in extraversion to AQ and -0.363 (*p* < 0.01) in perspective taking to AQ in non-ASD group whereas -0.402 (*p* < 0.01) in extraversion to AQ and -0.538 (*p* < 0.001) in perspective taking to AQ in ASD group, demonstrating that the test statistic for the difference between parameters were not significant (Supplementary Fig. S1).

## Discussion

The first finding of the present study is that ASD adults with no intellectual disability showed significantly lower scales for cognitive empathy, but not for affective empathy on the QCAE compared to control subjects. In subcategories of cognitive empathy, ASD subjects showed significantly lower scales for perspective taking and online simulation on the QCAE, and for perspective taking on the IRI compared to control subjects. In subcategories of affective empathy, ASD subjects showed lower scores for peripheral responsivity on the QCAE and empathic concern on the IRI than control subjects. Among these subscales, perspective taking and cognitive empathy on the QCAE demonstrated significant correlations with total AQ scores within each control group and ASD group. Furthermore, online simulation on the QCAE and perspective taking on the IRI showed significant correlations with AQ scores only within ASD group. The correlation plots for key results command visible comparison between the left control group and right ASD group (Fig. 1). Taken together, it is likely that the cognitive empathy scales as above are related with ASD traits. These findings suggest that autism is profoundly related to deficits in cognitive empathy, especially perspective-taking ability. In support, previous studies of empathy in ASD suggested that in adults with diagnoses such as Asperger syndrome, there were impairments on the perspective taking scale and deficits trends in the fantasy and empathic concern scales on the IRI^3,8–11^.

It is likely that cognitive and affective empathy are distinct categories. Cognitive empathy has been associated with the dorsomedial prefrontal cortex and midcingulate cortex, whereas affective empathy is linked to activity of the insula^28,29^. In the present study, ASD group showed significant alterations in the peripheral responsivity on the QCAE and the empathic concern on the IRI, but these scores did not show any significant correlations with AQ scores within each group. Additionally, ASD group failed to show changes in personal distress on the IRI. Incidentally, previous studies showed high scores for personal distress on the IRI in the Asperger syndrome and high-functioning ASD subjects^3,8,10,11^, but one study with no impairments^9^. It might be that deficits in affective empathy in individuals with ASD are difficult to check on the IRI or QCAE. Future study will be needed to elucidate this issue.

The second finding is that there existed contrasting patterns between the two group; significant correlations were seen between empathic concern on the IRI and proximal responsivity, peripheral responsivity and affective empathy on the QCAE only among control group, whereas a significant correlation was found between empathic concern on the IRI and emotional contagion on the QCAE only among ASD group. Further, there existed significant correlations between personal distress on the IRI and emotional contagion, proximal responsivity and affective empathy scores on the QCAE only among ASD group. These subscales are involved in affective empathy. It is natural that there exist significant associations between affective empathy-related subscales on the IRI and QCAE. However, it looks like a kind of shift from the empathic concern to the personal distress in ASD group because of reductions in scores for empathic concern and increases in scores for personal distress on the IRI. The pathway of affective empathy was divided into either empathic concern or personal distress from a view of responding. The tendency to feel compassion and concern for unfortunate others in empathic concern is in the opposite direction to the self-oriented feeling of anxiety and discomfort in response to other people's suffering in personal distress on the IRI. It is supposed that although ASD individuals can catch others' painful feeling, they are occupied by self-oriented feeling of distress (personal distress), making them difficult to have empathic concern to other peoples feeling (empathic concern) in affective empathy. This trade-off could be easily brought by the cognitive empathy deficits in case of ASD^3,8^. The group differences in peripheral responsivity on the QCAE and empathic concern on the IRI could support the importance of the correlations between peripheral responsivity on the QCAE and fantasy on the IRI and between empathic concern on the IRI and emotional contagion on the QCAE among ASD patients. Empathy has two directions to either voluntary behavior intended to benefit another or moral reasoning and social competence^30^. Helping behavior has two alternative motivations, selfless empathic concern in altruism and egoistic personal distress^31^. In another study, perspective taking in empathy needs self-awareness, mental flexibility and emotional regulation, and disturbed self-control process might induce personal distress^32^. Future study about affective empathy will be needed.

The third finding is that ASD adults with no intellectual disability had significantly higher scores for neuroticism and lower scores for extraversion on the NEO than control subjects after controlling for the full IQ and BDI scores by ANCOVA. AQ scores showed significant relationships with neuroticism, extraversion and conscientiousness on the NEO within control group and the combined total subjects but not ASD group. The correlation plots for key results command visible comparison between the left control group and right ASD group (Fig. 2). It looks that there exists a trend for characteristics of ASD traits when seen from a point of typical developing control. It might be due to the ceiling effects of NEO-PI-R on ASD subjects. In support of this, previous studies using a sample of typically-developing subjects, the five factor model of personality (FFM) accounted for 37% of AQ scores^33^, and the NEO-PI-R predicted 24% of the variability in AQ scores^34^. However, in other studies, the AQ scores were correlated with extraversion scale on the Eysenck Personality Questionnaire in the control adults and Asperger's syndrome group^35^, and all the scales on the FFM correlated with autism symptom severity on the Ritvo Autism/Asperger's Diagnostic Scale Revised^36^. It was unknown whether personality traits on the NEO could come from ASD traits on the AQ.

The fourth finding is that before Bonferroni correction, neuroticism on the NEO showed significant relationships with emotion contagion and affective empathy on the QCAE and fantasy on the IRI within respective two groups, and extraversion and openness on the NEO were significantly associated with peripheral responsivity on the QCAE within each group. Higher levels of neuroticism are associated with threat sensitivity and self-generated thought^37^. Further, within only ASD group, neuroticism on the NEO showed a significant relationship with personal distress on the IRI, and extraversion on the NEO showed significant relationships with online simulation and affective empathy on the QCAE. These indicated that there exist some relationships between personality components and empathy components. Further studies are required to address these results.

The final finding is that quantitative data analysis using multiple regression analysis with stepwise linear regression showed that two scores, perspective taking on the QCAE and extraversion on the NEO, were good predictor variables to autistic traits on the AQ scores. Although ASD individuals and non-ASD controls are different from a medical point of view, the above two factors in empathy and personality field could be significantly located in the pathophysiology of autistic traits in ASD.

### Limitations

This study has some limitations. First, sample sizes are small. Second, participants of two groups showed small differences in IQ between adults with ASD subjects and non-ASD controls in spite of recruiting participants with normal intelligence (full-scale IQ > 80). Third, ASD group showed depressive state despite that they were not suffering from depression. Fourth, there may be some issue using the total AQ score when assessing autistic traits^38^. Fifth, in a recent review study, the use of empathy measures did not show good psychometric properties in measuring empathy within an autistic population^39^. Sixth, it might be overestimating the correlation between the two measures on the QCAE and IRI because QCAE uses items from the IRI^25^. Seventh, about correlations between some empathy components on the IRI and QCAE, the contrasting patterns between the two groups were not statistically tested to be different through moderation analysis. Similarly, as for correlations between some empathy variables on the QCAE and IRI, and some components on the NEO, the contrasting patterns between the two groups were not statistically tested to be different through moderation analysis.

## Conclusions

ASD adults with no intellectual disability showed cognitive empathy deficits rather than affective empathy deficits, including perspective taking, online simulation and peripheral responsivity on the QCAE and perspective taking and empathic concern on the IRI, compared to non-ASD controls. Four scales, perspective taking on the QCAE and IRI, and online simulation and cognitive empathy on the QCAE, were significantly related with autistic traits on the AQ scores. ASD subjects showed higher scores for neuroticism and lower scores for extraversion on the NEO compared to non-ASD controls. However, these personality traits did not show any relationship with AQ scores in ASD subjects. Multiple regression analysis demonstrated that perspective taking score on the QCAE and extraversion score on the NEO were good predictor variables to autistic traits on the AQ scores. These results potentially help to explain what might underlie the empathy deficits and personality traits in people with ASD.

## Supplementary Information

Supplementary Information.

## Data Availability

The datasets are available from the corresponding author on reasonable request.

## Abbreviations

ADOS-2: The Autism Diagnostic Observation Schedule, 2nd edition
ANCOVA: Analysis of covariance
ASD: Autism Spectrum Disorder
AQ: Autism-Spectrum Quotient
BDI: Beck Depression Inventory
IQ: Intelligence Quatient
IRI: Interpersonal Reactivity Index
MANOVA: Multiple analysis of variance
NEO: NEO Personality Inventory-Revised
QCAE: Questionnaire of Cognitive and Affective Empathy
WAIS-III: Wechsler Adult Intelligence Scale, 3rd edition
