# Supplementary material for: Associations among autistic traits, cognitive and affective empathy, and personality traits in adults with autism spectrum disorder and no intellectual disability
Source: Sci Rep. 2022 Feb 24;12:3125. doi: 10.1038/s41598-022-07101-x (PMC8873508; doi:10.1038/s41598-022-07101-x)
Supplement: Supplementary file 1 — Supplementary Information. [file 41598_2022_7101_MOESM1_ESM.pdf]

	Cronbach's Alpha
AQ	.808
QCAE<br>Perspective taking	.922
QCAE<br>Online simulation	.894
QCAE<br>Emotion contagion	.715
QCAE<br>Proximal responsivity	.495
QCAE<br>Peripheral responsivity	.636
IRI<br>Perspective taking	.771
IRI<br>Empathic concern	.570
IRI<br>Personal distress	.782
IRI<br>Fantasy	.755
NEO<br>Neuroticism	.856
NEO<br>Extraversion	.820
NEO<br>Openness	.433
NEO<br>Agreeableness	.702
NEO<br>Conscientiousness	.807

Supplementary Table S1. Cronbach's Alpha of each scale in AQ, QCAE, IRI and NEO.

AQ: Autism-Spectrum Quotient. IRI: Interpersonal Reactivity Index. NEO: NEO Personality Inventory-Revised. QCAE: Questionnaire of Cognitive and Affective Empathy.

	Unstandardized coefficients B	Standardized coefficients Beta	*t* values	*p*-values	VIF
(Constant)	60.065 (2.951)		20.355	0.000 ***	
NEO Extraversion	-.430 (.058)	-.556	-7.430	0.000 ***	1.141
QCAE Perspective taking	-.573 (.085)	-.504	-6.746	0.000 ***	1.141

Excluded	*t* values	*p*-values	VIF
QCAE Online simulation	0.749	0.458	2.599
QCAE Emotion contagion	1.305	0.198	1.013
QCAE Proximal responsivity	1.359	0.180	1.136
QCAE Peripheral responsivity	0.415	0.680	1.507
IRI Perspective taking	-0.783	0.438	2.358
IRI Empathic concern	-0.050	0.960	1.113
IRI Personal distress	0.202	0.841	1.173
IRI Fantasy	0.303	0.763	1.107
NEO Neuroticism	2.841	0.007	1.189
NEO Openness	2.381	0.021	1.119
NEO Agreeableness	-0.604	0.549	1.053
NEO Conscientiousness	-0.062	0.951	1.320

Supplementary Table S2. Contributory factors to AQ scores by multiple regression analysis with stepwise linear regression method. B: multiple correlation coefficients. The number in the parenthesis is standard errors. η^2: Semi-partial eta-squared. Effect size represents a sample-based estimate of the quality. VIF: variance inflation factor. NEO: NEO Personality Inventory-Revised. QCAE: Questionnaire of Cognitive and Affective Empathy. ***$p < 0.001$.

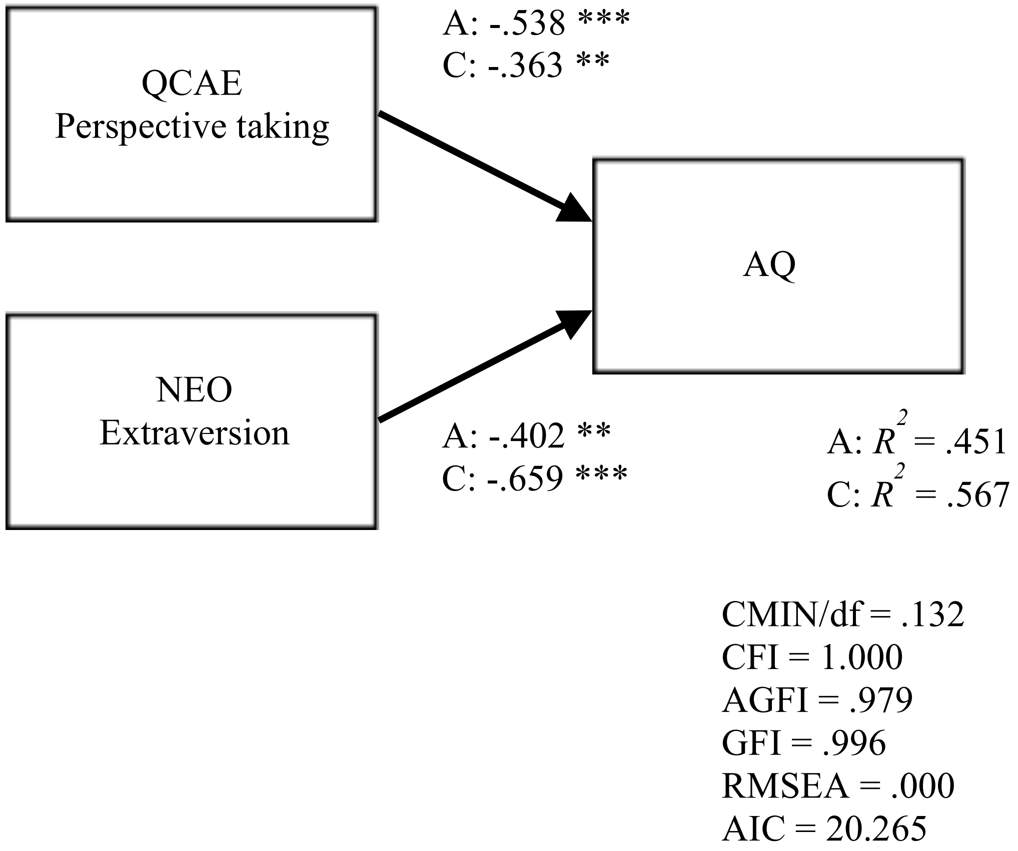

Supplementary Figure S1. Multiple group structural equation modeling: Comparison of the association between perspective taking, extraversion and autistic traits in people with or without ASD .

A: ASD group (n = 24). C: Non-ASD control group (n = 28). AQ: Autism-Spectrum Quotient. NEO: NEO Personality Inventory-Revised. QCAE: Questionnaire of Cognitive and Affective Empathy. Values with asterisk are standardized coefficients. $^{**}p < 0.01$, $^{***}p < 0.001$. R^2: R Square. CMIN: Chi-square statistics. CFI: Comparative Fit Index. AGFI: Adjusted Goodness-Fit Index. GFI: Goodness-Fit Index. RMSEA: Root Mean Square Error of Approximation. AIC: Akaike's Information Criterion.
